# PD-L1 Expression Fluctuates Concurrently with Cyclin D in Glioblastoma Cells

**DOI:** 10.3390/cells10092366

**Published:** 2021-09-09

**Authors:** Martina Tufano, Paolo D’Arrigo, Massimo D’Agostino, Carolina Giordano, Laura Marrone, Elena Cesaro, Maria Fiammetta Romano, Simona Romano

**Affiliations:** 1 Department of Molecular Medicine and Medical Biothecnology, University Federico II, 80131 Napoli, Italy; martina.tufano@unina.it (M.T.); massimo.dagostino@unina.it (M.D.); laura.marrone@unina.it (L.M.); elena.cesaro2@unina.it (E.C.); 2 Laboratory of Virology and Immunology, GIGA-R, B34, University of Liege, B-4000 Liege, Belgium; paolo.darrigo86@gmail.com; 3 Istituto di Neuroscience, Università Cattolica S. Cuore, 00168 Roma, Italy; carolinagiordano91@gmail.com

**Keywords:** PD-L1, Cyclin D, Glioblastoma, FKBP51

## Abstract

Despite Glioblastoma (GBM) frequently expressing programmed cell death ligand-1 (PD-L1), treatment with anti-programmed cell death-1 (PD1) has not yielded brilliant results. Intratumor variability of PD-L1 can impact determination accuracy. A previous study on mouse embryonic fibroblasts (MEFs) reported a role for cyclin-D in control of PD-L1 expression. Because tumor-cell growth within a cancer is highly heterogeneous, we looked at whether PD-L1 and its cochaperone FKBP51s were influenced by cell proliferation, using U251 and SF767 GBM-cell-lines. PD-L1 was measured by Western blot, flow cytometry, confocal-microscopy, quantitative PCR (qPCR), *CCND1* by qPCR, FKBP51s by Western blot and confocal-microscopy. Chromatin-Immunoprecipitation assay (xChIp) served to assess the DNA-binding of FKBP51 isoforms. In the course of cell culture, PD-L1 appeared to increase concomitantly to cyclin-D on G1/S transition, to decrease during exponential cell growth progressively. We calculated a correlation between *CCND1* and *PD-L1* gene expression levels. In the temporal window of *PD-L1* and *CCND1* peak, FKBP51s localized in ER. When cyclin-D declined, FKBP51s went nuclear. XChIp showed that FKBP51s binds *CCND1* gene in a closed-chromatin configuration. Our finding suggests that the dynamism of PD-L1 expression in GBM follows cyclin-D fluctuation and raises the hypothesis that FKBP51s might participate in the events that govern cyclin-D oscillation.

## 1. Introduction

PD-L1, also known as CD274 or B7-H1, is a transmembrane protein physiologically expressed on the plasma membrane of antigen presenting cells and aberrantly expressed by tumor cells, supporting tumor immune evasion [1]. Tumor PD-L1 expression can be constitutive or acquired [2]. Genetic/epigenetic alterations, deregulated activation of oncogenic signaling pathways and stimuli from microenvironment [2] are among the causes of aberrant expression of such an immune modulatory ligand in cancer cells. Especially, the epithelial to mesenchymal transition (EMT) state accounts for an acquired PD-L1 phenotype [3]. The EMT state attracts immune cells [3] that play a crucial role in stimulation of PD-L1 expression through IFN-γ and the type II IFN receptor signaling pathway [4].

PD-L1 is glycosylated on its route to plasma membrane, assisted by the isomerase activity of the splicing isoform of FKBP51 (FKBP51s) [5]. *FKBP5* is among the top 10% highly expressed genes in GBM (www.oncomine.org (accessed on 20 April 2019)); it correlates with glioma tumor grading [6] and supports the glioma stem cell niche [7]. The canonical FKBP51 protein structure includes a C-terminal TPR three-tandem-repeat domain responsible for protein–protein interaction, and two N-terminal FK domains, of which the first one exhibits peptidyl-prolyl cis-trans isomerase (PPIase) activity [8]. FKBP51s is generated by alternative splicing of *FKBP5* pre-mRNA, which causes a frameshift with a premature stop codon leading to a distinct C-terminus, compared to the canonical isoform [9]. FKBP51s retains the PPIase activity but loses the TPR domain. An IHC study on 29 GBM specimens showed that FKBP51s is broadly expressed in this tumor, albeit with different proportion/intensity scores, with nuclear and/or cytoplasmic localization [5].

Glioblastoma (GBM) is the most common primary malignant brain tumor, accounting for 54% of all gliomas and 16% of all primary brain tumors. Current treatment includes maximal safe resection, followed by radiotherapy with concurrent and adjuvant temozolomide [10]. The five-year survival is less than 5% after diagnosis, and the overall survival ranges from 12 to 18 months [10]. Currently, after surgery, the therapy remains difficult in that no contemporary treatments are curative. Results obtained with anti-PD1 therapy are below expectation, and in any case not as bright as those obtained for melanoma and lung cancer, despite GBM frequently expressing PD-L1 [11]. Evidence from randomized clinical trials suggest that only a few hypermutated glioblastoma represent an exception [12,13]. 

Heterogeneity of PD-L1 expression is common, partly responsible for some unreliable results on PD-L1 status [14]. Recently, Zhang et al. found that mouse embryonic fibroblasts (MEFs) KO for cyclin D1 had an increased level of PD-L1 [15]. The authors reported that PD-L1 protein abundance is regulated through the axis cyclin D/CDK4/Cullin3-SPOP that controls PD-L1 protein stability [15]. Cyclin D1-CDK4 directly phosphorylates SPOP at Ser6, which serves as an adaptor protein to Cullin3 ubiquitin ligase and promotes proteasome-mediated PD-L1 degradation [15].

Proliferation is an important part of cancer development and progression. Lack of normal growth control is not only operative in early tumorigenesis, but also during invasion and metastasis. The natural variability in the proliferative capacity of the cell population plays a relevant role in tumor evolution [16].

To provide more knowledge on PD-L1 expression in GBM, we investigated whether cyclin D and proliferation rates affected the expression levels of PD-L1 in GBM cell lines. At the same time, we investigated the subcellular distribution of FKBP51s during fluctuations of cyclin D that accompany cell cycle progression. Our study shows that PD-L1 expression is subjected to variations during GBM cell cultures. After cell seeding, we registered maximal expression before cell division concomitant to cyclin D peaking, and then it progressively decreased with cell growth, until cell confluency. FKBP51s cochaperone assists PD-L1 production in ER, and then it becomes nuclear when cyclin D declines.

## 2. Materials and Methods

### 2.1. Cell Cultures and Transfection

Human glioma cell lines U251MG and SF767MG were obtained and cultured as described [5]. The Dulbecco’s Modified Eagle Medium (DMEM) (Biowest, Nuaillé, France) without supplements was used for serum starvation assay. To investigate the effects of serum starvation, cells were initially grown for 4 h in the presence of FBS (Biowest) to allow cell adhesion to the plate, then the starvation lasted 32 hours before cell collection. For FKBP51s knockdown, 1 × 10^5^ cells were seeded on coverslips placed in 24-wells, 18 h before transfection, then, cells were transfected using the K2 Transfection System (Biontex, Munich, Germany), as previously described [5] in accordance with the manufacturer’s recommendations. FKBP51s silencing was performed using short-interfering oligoribonucleotides; the siFKBP51_1 silenced both *FKBP51* isoforms and was from Qiagen (Valencia, CA, USA) [9]; the siFKBP51_2 was a mix of 2 siRNAs designed on the coding-region, that selectively targets FKBP51s, as previously reported [9]. After a further 24 h from transfection, cells were used for the immunofluorescence staining, as described in the “Fluorescence Microscopy” paragraph. For overexpression of FKBP51 isoforms, shFKBP51.3 A375 cells were used as these cells are stably silenced for the *FKBP5* gene [17] and cultured, as previously described [17]. True-ORF-Myc-DDK-tagged expression vectors (OriGene Technologies, Rockville, MD, USA) carrying the cDNA of the human FKBP5 transcript variant 1 and 4 (FKBP51 and FKBP51s), respectively, were used. Control cells were transfected with the related empty vector (EV). After 24 h from transfection, cells were used for the quantitative PCR (qPCR) and chromatin immunoprecipitation (X-ChIP).

### 2.2. Cell Counting

Two ×10^5^ glioblastoma cells were seeded in 12-well plates and harvested at indicated times for cell count. Trypan Blue (diluted 1:10) was used to identify living cells that were counted by an optical microscope using the Bürker Chamber. For each well, 6 counts were performed. Three experiments have been performed in triplicates; means from the 3 experiments have been assessed for statistical analysis. 

### 2.3. Flow Cytometry

PD-L1 expression was measured using anti-B7H1-phycoerythrin (PE) (R&D Systems, Minneapolis, MN, USA) at a concentration of 0.05 μg/mL. A PE-conjugated control Ig isotype was used as control for non-specific binding. Briefly, cells were harvested and incubated with the antibodies for 30 min in the dark at 4 °C, washed and then analyzed by a BD Accuri™ C6 Cytometer (BD Biosciences, Franklin Lakes, NJ, USA).

### 2.4. Western Blot

Protein lysates were extracted using modified RIPA buffer and assayed by immunoblot as previously described [17]. The primary antibody against CD274/PD-L1, rabbit polyclonal (Novus Biological, Littleton, CO, USA) was used diluted 1:2500. Anti-G3PDH (rabbit monoclonal; Cell Signaling, Danvers, MA, USA) was used diluted at 1:1000. Anti-Histone H1 (1:500; AE-4, mouse monoclonal; Santa Cruz Biotechnology, Dallas, TX, USA) and anti-Calnexin (1:1000; AF18, mouse monoclonal; Invitrogen, Carlsbad, CA, USA) were used as nuclear and ER markers, respectively, while Coomassie Blue staining was used as a loading control.

### 2.5. Sub-Cellular Fractionation

Fractionation was performed as previously described [5]. Briefly, U251 MG glioblastoma cells were homogenized in 800 μL of Buffer F (0.25 M sucrose, 10 mM HEPES-NaOH, pH 7.2, 10 mM KAc, 1.5 mM MgAc) by pipetting the solution up and down 8–10 times through a 22-gauge needle. The nuclear fraction was sedimented by centrifugation for 5 min at 600× *g* and then resuspended in RIPA modified buffer. The post-nuclear supernatant was adjusted to 0.75 M sucrose, and the ER fraction was sedimented by ultracentrifugation for 12 min at 16,000 rpm and 4 °C in a Beckman with an SW 50.1 Ti rotor, and resuspended in RIPA modified buffer. Fractionated lysates were analyzed by immunoblot (see Western Blot section for antibodies specification).

### 2.6. Quantitative PCR (qPCR)

Total RNA was isolated from cells using Trizol (Invitrogen, Carlsbad, CA, USA) and 1 μg of each RNA was used for cDNA synthesis with iScript Reverse Transcriptase (Bio-Rad, Hercules, CA, USA). The reverse transcriptase reaction was performed as described [9]. Gene expression was quantified by qPCR with the 2^-^^ΔΔC^_T_ comparative method [18], using the SsoAdvancedTM SYBR Green Supermix (Bio-Rad) and specific qPCR primers to analyze each transcript. We performed relative quantitation of the transcript using a chosen control sample as expression = 1. Relative quantitation of the transcript was performed using co-amplified *ACTB*/ribosomal 18S as internal reference genes for normalization. For *PD-L1* [5], *CCND1* [17], and *ACTB*/18S rRNA [17] oligo sequences are used as previously described; for *CCNB1* and *CCND3*, specific real-time-validated QuantiTect primers from Qiagen were used.

### 2.7. Fluorescence Microscopy

Immunofluorescence staining was performed as previously reported [19]. Briefly, cells seeded on glass coverslips were collected after an 18h culture. For FKBP51s silencing experiments, cells were collected as described in “Cell Cultures and transfection”. Then, cells were washed with PBS and fixed with 3.7% formaldehyde (Sigma-Aldrich, St. Louis, MI, USA), at room temperature, for 30 min. After fixation, cells were washed with PBS and permeabilized by incubation in blocking buffer (PBS containing 1% BSA, 0.01% sodium azide and 0.02% Saponin) for 10 min at room temperature. Cells were then incubated with the indicated primary antibodies diluted in the same blocking buffer for 1 h at room temperature. Cells were washed three times with PBS and incubated with the corresponding secondary antibodies for 35 min at room temperature. The following antibodies were used: mouse monoclonal anti-CD107a (anti-Lamp1) #SAB4700416 (clone H4A3) from Sigma-Aldrich (St. Louis, MI, USA), rabbit polyclonal anti-Calnexin #SPC-108 from Stress Marq Biosciences Inc. (Victoria, BC V8N 4G0 Canada), rabbit polyclonal anti-FKBP51s (PCMR), mouse and rabbit Alexa-Fluor (488 and 546) secondary antibodies A11029, A11030, A11034, and A11035 (Thermo Fisher Scientific-Invitrogen, Carlsbad, CA, USA). Finally, coverslips were washed in distilled water and mounted onto glass slides with the Prolong Gold anti-fade reagent with DAPI (#P36935 Invitrogen, Waltham, MA, USA). Images were collected using a laser-scanning microscope (LSM 700, Carl Zeiss Microimaging, Inc., Jena, Germany) equipped with a planapo 63× oil-immersion (NA 1.4) objective lens. 

### 2.8. Chromatin Immunoprecipitation

A chromatin immunoprecipitation assay (X-ChIP) was performed as previously described [20]. Briefly, immunoprecipitated DNA and input controls were analyzed by qPCR using a SYBRGreen Master Mix (Bio-Rad) and the following primer pairs for *CCND1* promoter (-1564 → Fw: 5′-GGGCTGTCGGCGCAGTAGC-3′, Rev: 5′-GGTTACATGAGAGGGTCCCC-3′; -136 → Fw: 5′-GGGCTGTCGGCGCAGTAGC-3′, Rev: 5′-GCAGCACAGGAGCTGGTGTTCC-3′) and intronic (Fw: 5′-GAAAGTGCGGCGTGGTGCCC-3′, Rev: 5′-CCCTGAAAATGACCCTCGGGCG-3′) and flanking region (Fw: 5′-CCCCAGGTGCTCCCCTGACA-3′, Rev: 5′-CCCTCCTCCCCCACCGCT-3′). The enrichment percentage of DNA immunoprecipitated with anti-flag antibody (mouse monoclonal; Sigma-Aldrich) or with anti-trimethylated H3K4 (H3K4me3) and trimethylated H3K27 (H3K27me3) antibodies (rabbit polyclonal; Active Motif, Carlsbad, CA, USA) was calculated relative to the control IgG immunoprecipitated DNA. Histone modifications were then expressed as a H3K4me3/H3K27me3 ratio.

### 2.9. Statistical Analysis

The ANOVA assessed differences between means of values; a Sidak’s multiple comparisons test was used in case of multiple comparisons. These statistical analyses, along with linear regression analysis, were performed using Prism GraphPad 7.0a for Macintosh. A *p*-value ≤ 0.05 was considered statistically significant.

## 3. Results

### 3.1. Cell Growth Affects PD-L1 Expression

**Figure 1:**
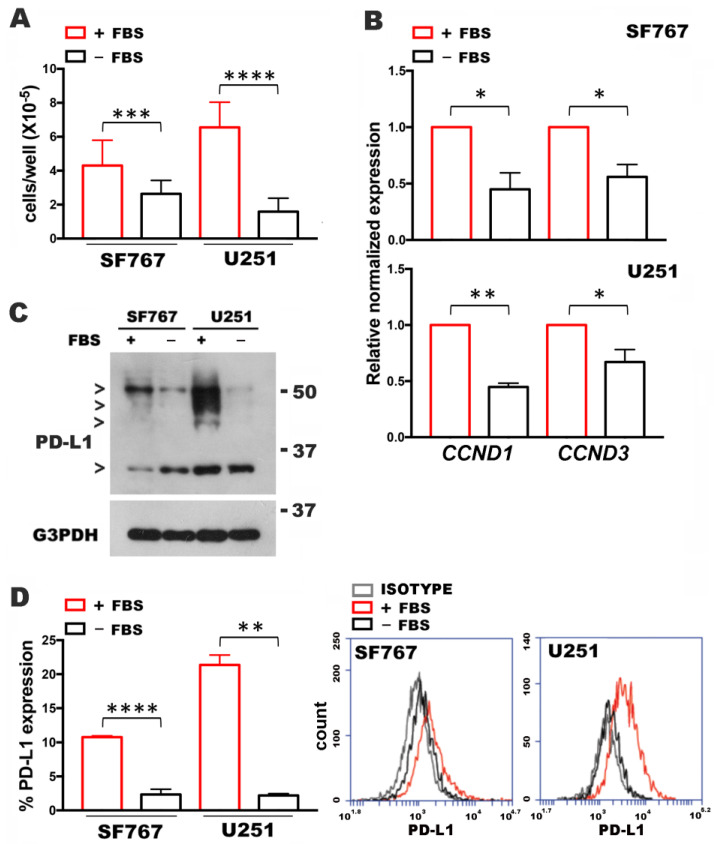
Effect of FBS deprivation on cyclin D and PD-L1 expression in U251MG and SF767MG cell lines. (**A**) Cell counting in cultures with or without FBS. Data are mean ± SD (N = 3). (**B**) Analysis by qPCR of *CCND1* and *CCND3* expression in condition of FBS depletion. (FBS+, control sample, expression = 1) (N = 3). (**C**) Western blot assay of PD-L1 expression in cultures with or without FBS. The band under 37kDa corresponds to naïve protein, bands near 50kDa correspond to glycosylated PD-L1. (**D**) Graphic representation of flow cytometric values (%) of PD-L1 expression. Data are mean ± SD (N = 3). On the left, representative histograms of expression are shown in overlay. * *p* < 0.05, ** *p* < 0.01, *** *p* < 0.001, **** *p* < 0.0001.

To address whether proliferative rates affected PD-L1 expression, we used U251MG and SF767MG glioblastoma cell lines, that constitutively express the immune-modulatory ligand [5]. In condition of serum starvation to slow proliferation, we analyzed the expression levels of cyclin D and PD-L1. Culture in medium without FBS produced a significant decrease in cell counts of both cell lines (from 4.3 ± 1.5 to 2.6 ± 0.7, and from 6.5 ± 1.4 to 1.5 ± 0.7, for SF767MG and U251MG, respectively) (Figure 1A). The measure of the transcript levels of cyclins D1 and D3 (Figure 1B) showed a significant decrease by serum deprivation. Assessment of PD-L1 expression by Western blot (Figure 1C) showed a band under 37 kDa, corresponding to naïve protein and bands around 40–50 kDa and to glycosylated PD-L1 [5]. PD-L1 expression was impaired by serum deprivation (Figure 1C). Such an impairment clearly relied on a glycosylated band (Figure 1C and Supplementary Information Figure S1). The reduced expression of glycosylated PD-L1 was confirmed by flow cytometry (Figure 1D) that measured a reduced plasma membrane expression of the ligand under serum starvation cultures (from 10.8 ± 0.2 to 2.3 ± 0.8, and from 21.3 ± 1.4 to 2.2 ± 0.2, for SF767MG and U251MG, respectively). The observation that naïve PD-L1 isoform was not affected (in U251MG) or even increased (in SF767MG) after serum deprivation (Figure S1) can be explained with protein stabilization according to a previous study [15]. However, the global impairment of mature protein expression suggests a more complex regulation of PD-L1 expression in GBM cells with slowed proliferation.

### 3.2. PD-L1 Levels Correlate with Cyclin D Levels

**Figure 2:**
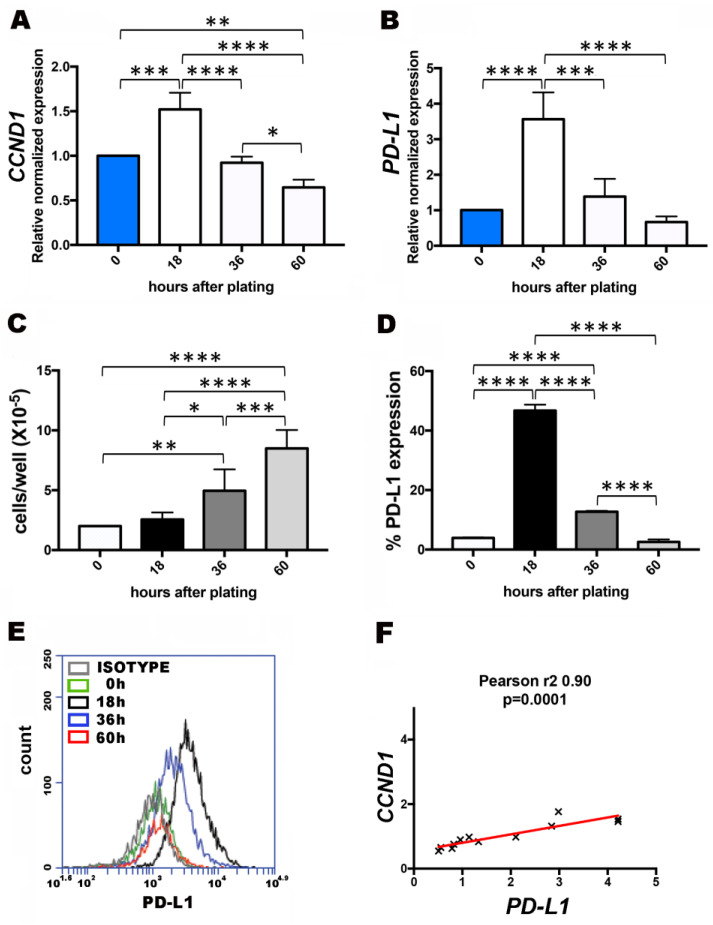
Changes in PD-L1 expression correlate with cyclin D expression, in U251MG cell culture. (**A**) Analysis by qPCR of *CCND1* expression level (T0 = control sample, expression = 1). (**B**) Analysis by qPCR of *CCND1* expression level (T0 = control sample, expression = 1). (**C**) Cell counting. (**D**) Flow cytometric analysis of PD-L1 expression. Cells were collected at 0h (seeding), 18 h, 36 h, 60 h after plating. (**E**) Representative histograms of PD-L1 expression are shown in overlay. (**F**) Linear regression of *PD-L1* and *CCND1* transcript levels (relative normalized expression, T0 reference sample). Data are mean ± SD (N = 3). * *p* < 0.05, ** *p* < 0.01, *** *p* < 0.001, **** *p* < 0.0001.

To address the dependence on cell growth of PD-L1 expression, we investigated whether PD-L1 levels changed during the phases of cell culture growth. After cell seeding, the growth of cells proceeds according to a standard pattern from the static phase to the log phase, where the cells proliferate exponentially. Then, cell contact slows cell proliferation. U251MG cells were collected at 0, 18, 36 and 60 h from cell seeding, counted and used partly for total RNA extraction and flow cytometry (Figure 2). As shown in Figure 2A,B, measure of *CCND1* and *PD-L1* transcripts showed maximal expression at 18 h, to progressively decrease with cell counts increase (Figure 2C). Cell counts (×10^−5^) were 2 ± 0, 2.5 ± 0.07, 4.9 ± 1.7, 8.5 ± 1.5, at 0, 18, 36 and 60 h, respectively (Figure 2C). By flow cytometry, we registered maximal PD-L1 expression after 18 h, (Figure 2D,E), to progressively decrease until 60 h. Values (%) of PD-L1 expression at 0, 18, 36 and 60 h were 3.9 ± 0.2, 46.7 ± 1.9, 19.7 ± 1.9, 2.5 ± 0.8 (Figure 2D). Figure 2E shows, in overlay, histograms of PD-L1 expression during the cell culture: the 18 h histogram (black) moved to the right of either 0 h (green), 36 h (blue), and 60 h (red) histograms, indicating increased expression. A linear correlation was calculated between *CCND1* and *PD-L1* gene expression levels (Figure 2F). Supplementary Information, Figure S2, shows a cell cycle analysis performed at the same time points. The maximal number of cells in S phase was registered at 36 h. Thus, the temporal window of 18h reasonably includes most of cells in G1-S transition. As cyclin D works at G1/S transition [21,22], the finding of *CCND1* peaking at this stage was expected. A similar trend of PD-L1 expression was observed in SF767MG cell line cultures (see Supplementary Information, Figure S3). 

### 3.3. FKBP51s Localizes in ER When CCND1 Peaks but in the Nucleus during Cell Division 

**Figure 3:**
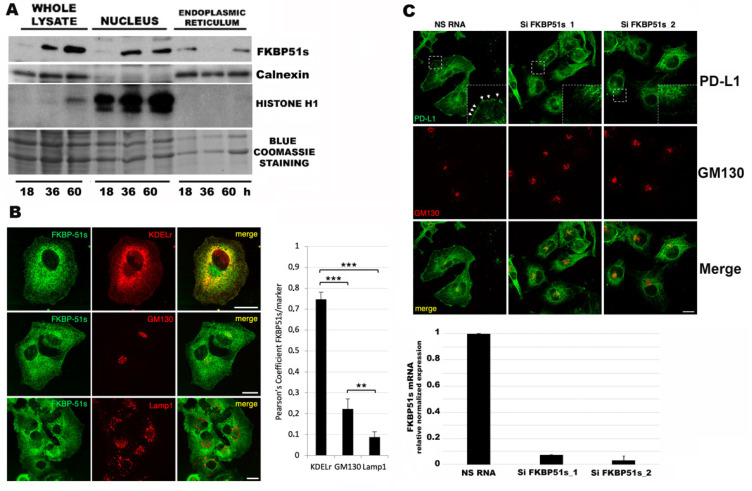
FKBP51s cellular distribution. (**A**) Western blot assay of total and fractionated (ER, nucleus) U251MG cell lysates. FKBP51s levels are shown along with relative organelle markers (calnexin, histone H1, for ER and nucleus, respectively) as loading controls. (**B**) Confocal microscopy: U251MG cells were seeded on coverslips and after 24 h were processed for indirect immunofluorescence by using specific antibodies against FKBP51s, KDEL receptor (ER marker), GM130 (Golgi marker), and Lamp1 (Lysosome marker). The histogram on the right indicates the colocalization (expressed with Pearson’s Coefficient) of FKBP51s with different markers of the intracellular compartments. Scale bar, 50 um. Data are mean ± SD (N = 3), ** *p* < 0.01, *** *p* < 0.001. (**C**) Twenty-four h after transfection with NS RNA or SiFKBP51s, U251MG cells (seeded on coverslips) were handled as in B immunofluorescence by using specific antibodies against GM130 (Golgi marker) and PD-L1 (Upper). Zoomed cropped insets are shown in the dashed white squares. White arrows indicate the plasma membrane localization of PD-L1. Single focal sections are shown. Scale bar, 50 um. Analysis by qPCR of *FKBP51s* transcript levels in U251 transfected cells (relative normalized expression, NS RNA reference sample) is also shown (Lower). Data are mean ± SD (N = 4).

In a previous paper, immunohistochemistry of FKBP51s found this protein variably expressed in the cytoplasm and/or nucleus of tumor cells in patient GBM specimens [5]. Because FKBP51s interacts with PD-L1 in ER assisting protein maturation [5], we looked at its localization during cell culture, in an attempt to clarify whether this protein was present in ER in the temporal window of PD-L1 upregulation. Protein was extracted from the whole cell, ER and nucleus of U251MG cells at 18 h, 36 h and 60 h, and the extracts were run in Western blot. Levels of FKBP51s in whole lysates appeared to increase from 18 to 60 h (Figure 3A). At 18 h, FKBP51s appeared in ER. At 36 h, an increased protein level was measured in the nucleus; at 60 h, FKBP51s was present in both compartments. Confocal microscopy analysis confirmed ER localization of FKBP51s (Figure 3B). Indeed, using probes for ER (KDELr), Golgi (GM130) and lysosome (Lamp1), we could assess the predominant distribution of FKBP51s in the ER compartment. Confocal microscopy (Figure 3C) showed that PD-L1 was retained in the ER when cells were depleted of FKBP51s and cannot progress to the Golgi and plasma membrane. As shown in Figure 3C, PD-L1 is expressed on the plasma membrane (see also enlarged detail) and in Golgi (yellow, in Merge panel) of U251MG cells treated with a non-silencing RNA (NS RNA). PD-L1 disappeared from the plasma membrane (see also enlarged detail) and its expression in Golgi decreased (orange/red, in Merge panel of Si FKBP51s cells) in U251MG cells treated with two different FKBP51s silencing RNAs (Si FKBP51s). 

### 3.4. FKBP51s Interacts with CCND1 Promoter in a Closed Chromatin State

**Figure 4:**
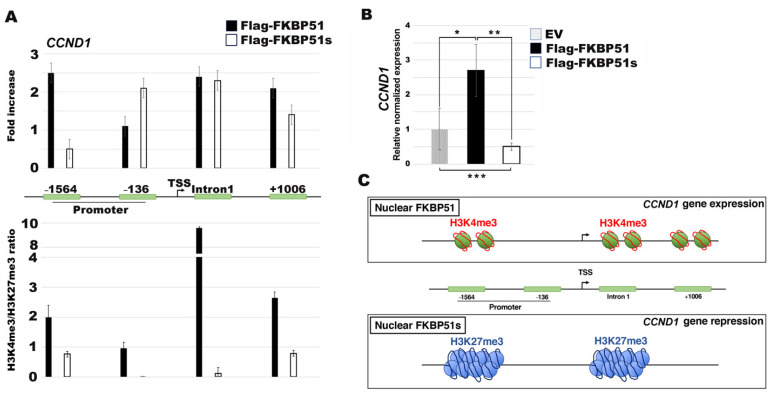
FKBP51 isoforms occupancy on the *CCND1* promoter. (**A**) Upper panel, xChIp assay performed with an anti-Flag antibody (upper panel) and with anti-H3K4me3 and anti-H3K27me3 (lower panel) in shFKBP51.3 A375 cells overexpressing Flag-FKBP51 or Flag-FKBP51s. After 24 h from transfection, qPCR analysis was performed using primers covering the 4 represented regions of the *CCND1* gene (−1564 and −136 in the promoter and intron 1 and +1006 in the intronic sequences following the TSS). (**B**) QPCR analysis of *CCND1* mRNA expression in shFKBP51.3 A375 cells, transfected with empty vector (EV, grey) or the FKBP51 isoforms (Flag-FKBP51, black; or Flag-FKBP51s, white). (EV = control sample, expression = 1). Data are mean ± SD (N = 4). * *p* = 0.014, ** *p* = 0.01, *** *p* = 0.032. (**C**) Schematic representation of the chromatin status on the *CCND1* gene in cells overexpressing FKBP51 or FKBP51s.

To investigate the role of FKBP51s in the nucleus, we performed an X-ChIP assay. To this end, we employed a melanoma FKBP51 knocked-down cell line (shFKBP51.3 A375 cells), as previously generated [17]. We overexpressed FKBP51s or the canonical FKBP51 for comparison (Supplementary Information, Figure S4). Chromatin was immunoprecipitated with a specific antibody recognizing the Flag tag and then analyzed by qPCR using oligonucleotides covering both the *CCND1* promoter and intronic sequences closer to the TSS site. Our results show both FKBP51 isoforms occupancy of the *CCND1* promoter and intronic regions (Figure 4A, upper). We then interrogated the *CCND1* promoter and intronic sequences for their H3K27me3 and H3K4me3 pattern in shFKBP51.3 cells expressing FKBP51 isoforms. Our data revealed H3K4me3 modifications, associated with higher transcription activity, occurring mainly in Flag-FKBP51 immunoprecipitated chromatin to the detriment of H3K27me3, thus producing a ratio of α-H3K4me3/α-H3K27me3 >1 only in FKBP51, but not FKBP51s overexpressing cells (Figure 4A, lower). In line with this finding, *CCND1* mRNA levels were significantly reduced in FKBP51s overexpressing cells, compared to EV levels (Figure 4B). In contrast, in FKBP51 overexpressing cells, *CCND1* mRNA levels were significantly increased compared to EV levels (Figure 4B). Figure 4C depicts a schematic representation of the chromatin status on the *CCND1* gene in cells overexpressing FKBP51 or FKBP51s. Data were confirmed in another independent experiment. A further investigation was performed, employing an FKBP51 mutant harboring a point mutation of the TPR domain (Flag-FKBP51-mutTPR) which is the domain absent in FKBP51s [17] (Supplemental Information Figure S5). Similar to FKBP51s, the FKBP51-mutTPR was also found to be associated with the *CCND1* promoter. The ratio H3K4me3/H3K27me3, even still >1, appeared to be reduced compared to the FKBP51 ratio. This finding reinforces the role of FKBP51 in the open chromatin status. 

## 4. Discussion

Intra-tumor heterogeneity of PD-L1 expression is common, variable in scale and extent, and can determine inaccurate stratification of patients to immunotherapy [23,24]. In glioblastoma tumors, high PD-L1 expression is associated with poor patient survival [25]. PD-L1 is not only a prognostic biomarker of immune therapy, but also a potential therapeutic target for glioblastoma [26], therefore, the unfavorable outcomes of this incurable tumor can significantly benefit from understanding the complex dynamics underlying PD-L1 expression regulation. 

Here, we show a temporal-dependent mechanism of PD-L1 expression modulation in GBM cells. Our finding shows changes of PD-L1 expression levels during GBM cell culture growth. More precisely, an increase in PD-L1 was registered very early after cell seeding, coinciding with the increase of cyclin D expression to progressively decrease as the cells divide and grow, until cell confluency, where we registered the lowest level. We found a correlation between *CCND1* and *PD-L1* expression levels, suggesting such genes are contextually transcribed in a temporal window during cell cycle progression. Moreover, the *PD-L1* mRNA increase coincided with increased protein expression. Within the same temporal window, the PD-L1 cochaperone, FKBP51s, appeared in the ER to assist protein production. When cell division and growth progressed, PD-L1 transcription declined along with the protein level. 

A previous study reported oscillation of PD-L1 expression in MEFs during cell cycle progression. The authors demonstrated a role for cyclin D in controlling PD-L1 protein abundance using a genetic method to ablate cyclins in MEFs [15]. The effect of cyclin D1-CDK4-SPOP-Cullin3 axis on the destabilization of PD-L1 protein [15] together with the observed reduced gene expression could lend support to the decrease in expression of the immunomodulatory molecule that occurred as soon as after cyclin D peaking. Interestingly, PD-L1 decrease was accompanied by the appearance of FKBP51s in the nucleus. Even if the role of this protein in the nucleus remains unknown, we found that FKBP51s binds the promoter of *CCND1* in a closed chromatin configuration, suggesting it can take part in the events that regulate the cyclic expression of *CCND1* during cell cycle progression. Such a hypothesis deserves to be investigated in the future. Additionally, studies on patient biospecimens are required to validate the results obtained with the cell lines.

In conclusion, our study provides novel elements that link PD-L1 expression to cell proliferation. A relationship between PD-L1 expression and proliferation was firstly reported by Xue et al., who performed correlative studies of protein profiles of PD-L1 and KI-67 in glioma patients [27]. The natural variability in the proliferative capacity observed among cells of a single tumor significantly affects tumor progression and therapeutic effectiveness [14]. The relationship between PD-L1 and cell proliferation carries important implications on the possible linkage between heterogeneity in the cell cycle duration and heterogeneity of PD-L1 expression in GBM tumors. Given the foregoing, our findings can open the door to novel strategies to target PD-L1, acting on cell cycle regulators. In this regard, the use of cell synchronization agents can help to create an optimal window for checkpoint-targeted immunotherapy.

It should be noted that, in addition to PD-L1, another PD1 ligand, the programmed cell death ligand-2 (PD-L2/CD274) is emerging as clinically relevant in primary brain tumors [28,29]. The finding that PD-L2 expression correlated with worse clinical outcomes in low- and high-grade glioma supports possible treatment options with anti-PD-L2 in GBM patients [28,29]. Fu et al. observed high constitutive expression of PD-L2 along with PD-L1 in a subset of brain tumor cell lines and patient-derived brain tumor-initiating cells [29]. The authors found regulatory regions under GATA2 control in both PD-L1 and PD-L2 genes [29], suggesting common mechanisms regulated tumor cell-intrinsic expression of the two PD1 ligands. Unlike PD-L1, PD-L2 remains poorly explored; whether its expression is subjected to cell-cycle-related fluctuations deserves to be addressed in future studies.

## Data Availability

The authors confirm that the data supporting the findings of this study are available within the article and its Supplementary Materials.

## Supplementary Materials

The following are available online at https://www.mdpi.com/article/10.3390/cells10092366/s1, Figure S1: Densitometry analysis of bands in Western blot of Figure 1D, Figure S2: Cell cycle analysis in the course of U251MG cell culture, Figure S3: Changes in PD-L1 expression during SF767MG cell culture, Figure S4: Western blot assay of FKBP51-KO-A375 cells transfected with the FKBP51 isoforms, Figure S5: Mutated TPR domain of FKBP51 affects occupancy of the *CCND1* promoter.
