# Supplementary material for: PD-L1 Expression Fluctuates Concurrently with Cyclin D in Glioblastoma Cells
Source: Cells. 2021 Sep 9;10(9):2366. doi: 10.3390/cells10092366 (PMC8468141; doi:10.3390/cells10092366)
Supplement: Supplementary file 1 [file cells-10-02366-s001.zip › cells-1340341-supplementary.pdf]

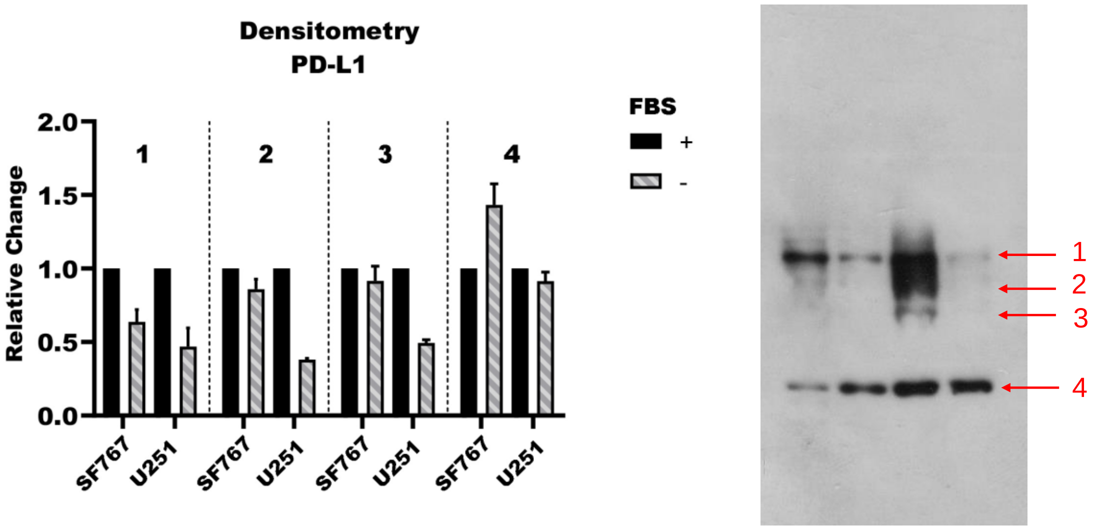

Figure S1 *Densitometry analysis of bands in Western blot of Fig 1 C.* Quantification of bands was obtained by densitometry analysis using ImageJ 1.42q for Macintosh. Integrated OD was normalized to G3PDH and expressed as fold increase, using expression in FBS+ culture as the reference sample (=1). Data are mean ± SD (N=3).

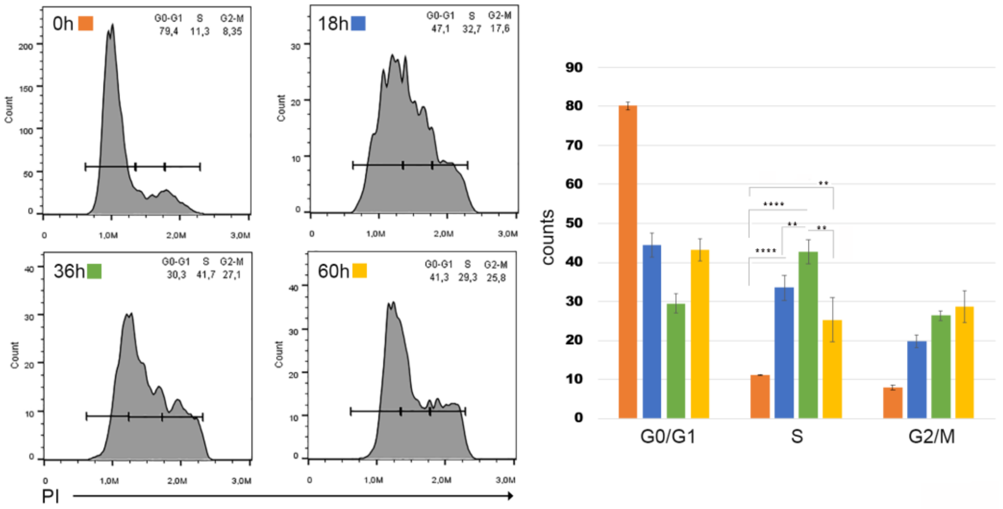

Figure S2 *Cell cycle analysis in the course of U251MG cell culture.* On the left, representative flow cytometric histograms; on the right, graphic representation of G0/G1, S and G2/M values (mean ± SD, N=3). Briefly, U251MG cells were collected at 0h (seeding), 18h, 36h, 60h after plating and washed in PBS. Then, cells were fixed in cold 70% ethanol, added drop wise to the pellet while vortexing to ensure fixation of all cells and minimize clumping, for 2h at 4°C. Cells were then washed twice in PBS, treated with ribonuclease and stained with 200 µl PI (from 50 µg/ml stock solution). After 20 min in the dark at 4 °C, samples were analyzed by a BD Accuri™ C6 Cytometer (BD Biosciences, New Jersey, USA). FlowJo software was used for the analysis. Data are mean ± SD (N=4). *P<0.05, **P<0.01, ***P<0.001, ****P<0.0001.

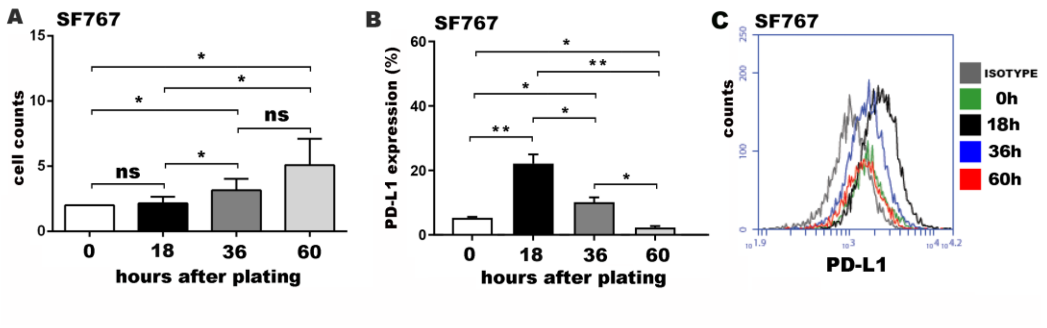

Figure S3 *Changes in PD-L1 expression during SF767MG cell culture*. (A) Cell counting and (B) flow cytometric analysis of PD-L1 expression. Cell counts ($x10^{-5}$) measured at 0, 18, 36 and 60h from cell seeding were 2±0, 2.1±0.5, 3.1±0.8, 5.1±2. Values (%) of PD-L1 expression at 0h, 18, 36 and 60h were 5.2±0.3, 22.3±2.8, 10.1±1.5, 2.2.±0.4 Data are mean ± SD (N=3). *P<0.05, **P<0.01, ***P<0.001, ****P<0.0001. (C) Representative histograms of PD-L1 expression are shown in overlay.

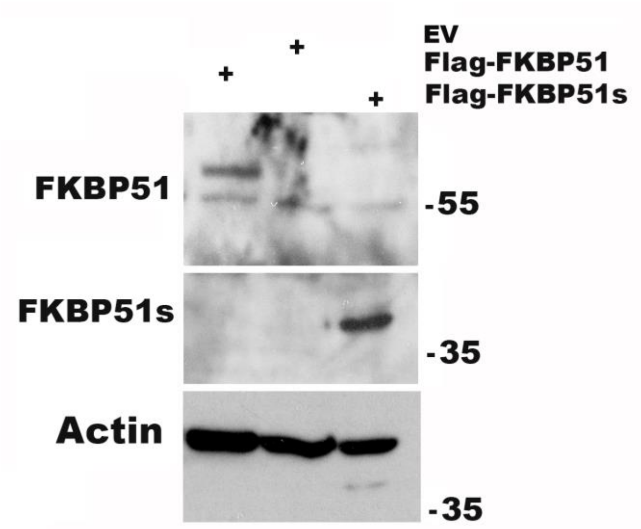

Figure S4 Western blot assay of FKBP51-KO-A375 cells transfected with the FKBP51 isoforms

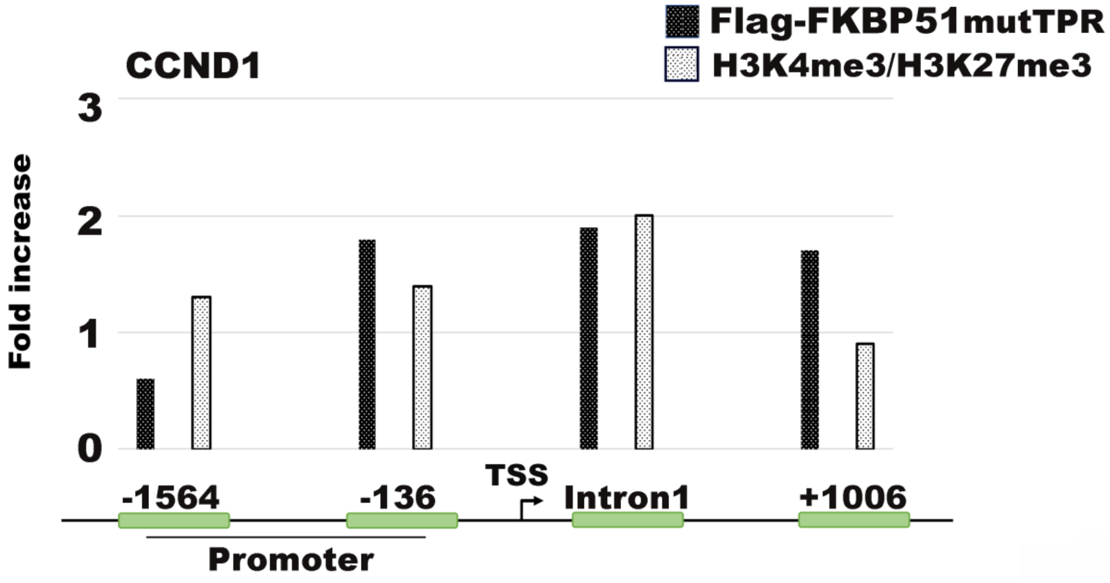

Figure S5 *Mutated TPR domain of FKBP51 affects occupancy of the CCND1 promoter*. X-ChIP assay was performed with an anti-Flag antibody (upper panel) and with anti-H3K4me3 and anti-H3K27me3 (lower panel) in shFKBP51.3 A375 cells overexpressing Flag-FKBP51nutTPR. After 24 hours from transfection, qPCR analysis was performed using primers covering the 4 represented regions of the *CCND1* gene (-1564 and -136 in the promoter and intron 1 and +1006 in the intronic sequences following the TSS).
